# More than a Pretty Place: Assessing the Impact of Environmental Education on Children’s Knowledge and Attitudes about Outdoor Play in Nature

**DOI:** 10.3390/ijerph120202054

**Published:** 2015-02-12

**Authors:** Kirsten M. M. Beyer, Elizabeth F. Heller, Jessica M. Bizub, Amy J. Kistner, Aniko Szabo, Erin E. Shawgo, Corey J. Zetts

**Affiliations:** 1 Division of Epidemiology, Institute for Health and Society, Medical College of Wisconsin, 8701 Watertown Plank Road, Milwaukee, WI 53226, USA; 2 Urban Ecology Center, 1500 East Park Place, Milwaukee, WI 53211, USA; E-Mails: bheller@urbanecologycenter.org (E.F.H.); Erin.shawgo@gmail.com (E.E.S.); 3 Center for Urban Initiatives and Research, University of Wisconsin-Milwaukee, Engelmann Hall, B59, P.O. Box 413, Milwaukee, WI 53201, USA; E-Mail: bizub@uwm.edu; 4 Department of Family and Community Medicine, Medical College of Wisconsin, 1155 North Mayfair Road, Milwaukee, WI 53226, USA; E-Mail: akistner@mcw.edu; 5 Department of Psychiatry, Medical College of Wisconsin Tosa Center, 1155 Mayfair Road, Milwaukee, WI 53226, USA; 6 Division of Biostatistics, Institute for Health and Society, Medical College of Wisconsin, 8701 Watertown Plank Road, Milwaukee, WI 53226, USA; E-Mail: aszabo@mcw.edu; 7 Menomonee Valley Partners, Inc., 301 W. Wisconsin Ave., Suite 400B, Milwaukee, WI 53203, USA; E-Mail: Corey@RenewTheValley.org

**Keywords:** urban environmental education, children’s outdoor play, barriers to nature, fear, ATOP scales

## Abstract

Our work assessed the influence of an urban environmental education program on children’s attitudes toward outdoor play, as well as knowledge of neighborhood features that can facilitate this type of activity. The project team engaged 6 schools near the newest Urban Ecology Center location in Milwaukee, Wisconsin, USA, through a community-academic partnership entitled More Than a Pretty Place. Intervention classrooms participated in programming over the 2012–2013 academic year and pre and post surveys were implemented in classrooms. Data were analyzed using multilevel regression models. The intervention group reported reduced fears of outdoor play in nature and increased frequency of visits to the Urban Ecology Center. The proportion of students who acknowledged knowing of a place to play outside in nature increased significantly in both groups. Our findings indicate an important role for environmental education in addressing fears that may dissuade children from engaging in outdoor play in natural areas.

## 1. Introduction

Physical activity is associated with a wide range of physical and mental health benefits, including weight control, metabolic health, and reductions in depression, anxiety and stress. Increasing levels of physical activity is a prime target for reducing the ill effects of overweight and obesity and for preventing and controlling chronic diseases, including several types of cancer, diabetes and cardiovascular diseases [1]. Physical activity during childhood and adolescence is one of the best predictors of adult physical activity, and evidence has shown that promoting and establishing lifestyles that incorporate physical activity among children is often more effective and easier than promoting physical activity among adults [1]. In addition, physical activity confers numerous immediate benefits for children. A recent systematic review found benefits for overweight/obesity, blood pressure, bone strength, aerobic fitness, strength and endurance, depression, anxiety, and several measures of self-concept among children and youth engaging in physical activity [2]. Overall, there is little doubt that increases in physical activity will lead to improvements in physical and mental health among children and adults. 

Recent work has shown that undertaking physical activity in outdoor, natural environments may provide added benefits [3,4]. Pretty *et al.* found that individuals exposed to scenes of greenery while exercising on a treadmill experienced significant reductions in blood pressure and improvements in mood, when compared to subjects undertaking exercise without exposure to such images [4]. Barton and Pretty found in a meta-analysis that acute short-term (5 min) exposures to green exercise (activity in the presence of nature) improved both self-esteem and mood for a variety of green environments, with a higher effect size observed for activities in the presence of surface water [3]. Hug *et al.* found that fitness center members in Zurich identified activities in outdoor settings as more restorative than those undertaken indoors, and that perceived restorative quality was a predictor of exercise frequency [5], which could improve benefit through increased engagement in physical activity. Mitchell found in a population-based survey study in Scotland that the odds of poor mental health were reduced among those regularly using natural settings for physical activity, compared to non-users of these settings [6]. A recent systematic review found outdoor exercise in natural environments to be associated with increased feelings of revitalization, positive engagement, and energy, and decreases in tension, confusion, anger and depression [7]. These study findings are further supported by a growing body of evidence pointing to many benefits of access to, knowledge of and engagement with green space and nature, including increased numbers of social contacts and levels of social cohesion [8,9,10,11], and mental health benefits such as reduced stress [12,13,14,15] and reduced mental fatigue [16,17,18,19,20,21,22]. In addition, physical activity may enhance the restorative benefit of exposure to nature [23].

One category of activity of growing interest is active outdoor play among children. *Last Child in the Woods*, a seminal work by Richard Louv, brings together numerous studies that point to direct exposure to nature as essential for a child’s healthy physical and emotional development [24]. Studies have linked outdoor activities, particularly unstructured outdoor play in natural environments, to improvements in motor skill development [25] as well as problem-solving skills, social relationships, and emotional well-being [26]. Fjørtoft found in a quasi-experimental study in Norway that an experimental group of children offered unstructured play time in nature showed greater improvements in motor skills as measured by the EUROFIT Motor Fitness Test than a control group playing in their normal outdoor play setting [25]. In addition, a study of children with Attention Deficit Hyperactivity Disorder (ADHD) found mental health benefits of outdoor walks in a natural setting, as compared to a downtown or neighborhood setting, with improvements in concentration comparable to improvements gained by commonly prescribed pharmaceuticals for ADHD [16]. Similarly, Faber-Taylor and Kuo found in a national study that children’s ADHD symptoms were milder if they played regularly in green settings [27], providing additional support for the mental health benefits of outdoor play and physical activity among children.

Because children’s physical activity is of immediate benefit, predicts adult physical activity, and is often easier to effect than adult physical activity [1], encouragement of physical activity among children—and outdoor physical activity in particular, given its added benefits—is a promising target for public health intervention. However, at the same time that the body of evidence proclaiming the benefits of physical activity, including outdoor activity, accumulates, there is a growing concern that children’s engagement with the natural world, particularly through active outdoor play, has decreased over time [24,28]. Researchers point to a number of barriers that can prevent children from engaging in outdoor play in nature, particularly in urban settings. Gaining access to nearby natural areas can be challenging, particularly for racial and ethnic minority children in urban environments where green spaces are less prevalent [29]. In addition, parental concerns about safety can limit children’s exposure to and knowledge of natural environments such as urban parks [30,31,32], and aspects of the built environment, including the nature of the urban transportation system and resulting hazards and potential exposures, can serve as barriers to children’s outdoor play and physical activity [33]. Because of the nature of these barriers, the result may be that low socio-economic status (SES), urban children—often children of color—engage in less outdoor play. In our study area of Milwaukee, Wisconsin, USA, lower SES groups report lower rates of physical activity [34]. Other studies have found reduced activity among low SES and racial and ethnic minority groups [35].

As the benefits of active outdoor play become more apparent and concern rises that children are becoming disconnected from those benefits, identifying and overcoming barriers to children’s active outdoor play, particularly in urban environments, becomes critical. Strategies are needed to facilitate children’s engagement in these activities to ensure they receive the known health benefits of active outdoor play. 

One approach to connecting children with natural areas is environmental education. With a goal and long history of engaging individuals in programming to facilitate the development of environmental literacy, as well as changes in knowledge, skills and behaviors to ultimately improve environmental health and sustainability [36], environmental education is a promising approach to connecting children with nature to improve their social, emotional, physical and intellectual health, and may be especially important in urban settings where natural areas are more challenging to access. Particularly at a time when conversations about evaluation approaches for environmental education programming are fresh in memory [37], additional consideration of the potential physical and mental health benefits of environmental education programming are appropriate and timely.

Health behavior theory would suggest that a primary target for intervention to ultimately increase children’s engagement in active outdoor play is the set of health beliefs, attitudes and knowledge that govern that behavior [38]. Thus, the objective of this work is to assess the influence of participation in an urban environmental education program on children’s attitudes toward outdoor play, as well as their knowledge of and engagement with neighborhood features that can facilitate this type of activity.

## 2. Methods

### 2.1. More than a Pretty Place: Overview

More Than a Pretty Place is a community-academic partnership project funded by the Healthier Wisconsin Partnership Program (2012–2014) in the city of Milwaukee with a goal of identifying the health benefits of nature-based outdoor recreation and exploration. Project partners include the Urban Ecology Center, Menomonee Valley Partners, Inc. and researchers at the University of Wisconsin-Milwaukee and the Medical College of Wisconsin.

The Urban Ecology Center (http://urbanecologycenter.org/) is a Milwaukee-based nonprofit organization dedicated to fostering ecological understanding as inspiration for change, neighborhood by neighborhood. Staff at three Urban Ecology Center locations provide outdoor environmental and science education for urban youth, contribute to protecting and using public natural areas, serve as models for environmentally responsible behaviors, and offer resources to neighborhood residents to support learning, volunteerism, land stewardship, recreation and camaraderie. Menomonee Valley Partners (http://www.renewthevalley.org/) is a community development organization that has led the redevelopment of Milwaukee’s Menomonee Valley, a former industrial zone including 300 acres of brownfields.

In 2002, Menomonee Valley Partners and the Urban Ecology Center decided to combine the best of both organizations and undertake a series of interdependent projects to connect communities to jobs, environmental education, restored natural resources and new recreational opportunities. This joint effort, entitled Menomonee Valley‐From the Ground Up (http://www.renewthevalley.org/documents/18-menomonee-valley-from-the-ground-up), was designed to revitalize Milwaukee neighborhoods hit hardest by the Menomonee Valley’s economic decline. Elements of the project include: (1) Expanding the Hank Aaron State Trail by 6 miles to create an east‐west bike/pedestrian corridor to serve the region’s commuter and recreational needs, (2) Establishing a new Urban Ecology Center branch (opened in September 2012) that offers educational and recreational programming and acts as the primary land steward, and (3) Transforming a vacant rail yard into a 24‐acre park to be the new center’s outdoor classroom while improving ecological conditions and providing green space for neighborhood residents. In 2011, the Urban Ecology Center and Menomonee Valley Partners decided that the new branch location provided a rare opportunity to evaluate the community health impacts of their work, as a cohort of students from neighborhood schools would be enrolled in neighborhood-based environmental education programming for which they were not previously eligible. They reached out to researchers at local academic organizations, and a partnership developed. The More Than a Pretty Place project began formally in January of 2012.

### 2.2. The Neighborhood Environmental Education Project (NEEP)

**Figure 1:**
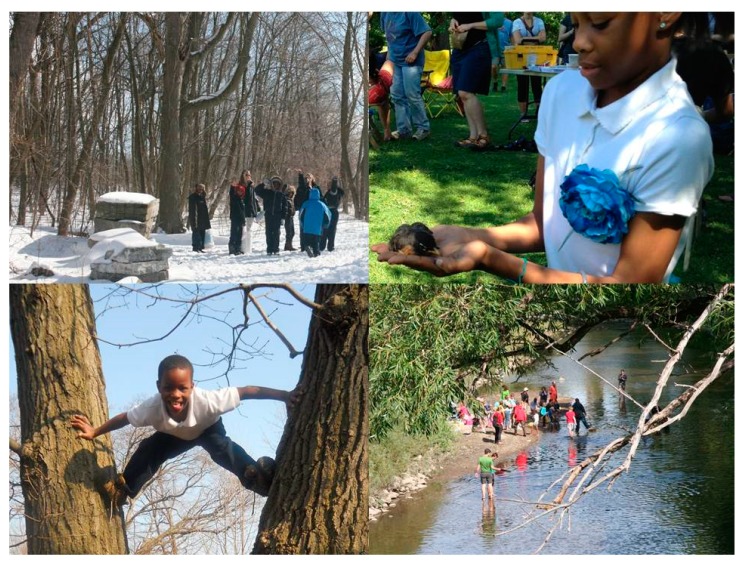
Children engaged in NEEP programming (pictured are not study subjects).

The Urban Ecology Center offers, for a fee, neighborhood environmental education project (NEEP) programming to schools within a 2-mile radius of each of its three Milwaukee locations. These concentrated focus areas are designed to make it possible for NEEP participants to visit the Urban Ecology Center on their own time, even with limited access to reliable transportation. NEEP participants engage in a variety of activities (see Figure 1) that are adapted for the parks adjacent to each of the three Urban Ecology Center branches.

In the Menomonee Valley, NEEP programming examined in this study consisted of hands-on science education for K-12 students. Education activities are aligned to science standards, including Wisconsin State and Next Generation Science Standards, and focus on nature as a context for learning. Schools sign up for an average of 24 field trips and distribute those between classrooms, resulting in an average number of visits per student of just fewer than three times per year. The annual contract renewal rate for schools is 95%, meaning students are very likely to return for multiple years. Therefore, the course offerings vary by grade, offering a unique experience for students every time they visit the Center. For this study, students were attending for the first time because this was the first year the Center was open.

Fifth and sixth grade programs included the following: “Where am I” (students walk through the park, mapping natural features), “Water, Water Everywhere” (through instructor led demonstrations and games, students explore concepts of watersheds, hydrology, accumulation and percolation, then study the land for evidence of these concepts), “Sustainable Food” (through discussion and reflection, students learn about sources of food for people, then study food chains in nature and how to grow their own food), “Winter Ecology” (students play games to understand how plants and animals are adapted for winter conditions, then find examples of adaptations in the natural areas), “Carrying Capacity and Extinction,” (through simulations, graphing and play, student learn about ecosystem dynamics and carrying capacity; outdoors students collect data and make inferences about the carrying capacity of urban parks) “Recycling,” (students build a deeper understanding of recycling and then study how organisms in an urban park recycle nutrients), “Nature Journaling and Phenology,” (students make observations, and infer changes over time in the park; they write and draw what they learn) “Life Cycles,” (while exploring the outdoors, students look for evidence of natural cycles, such a plant reproduction, the water cycle, or nutrient cycles) “Birds of a Feather,” (using binoculars, students learn concepts of ornithology and adaptation as they identify distinguishing characteristics and behaviors of local birds) “Microscopes and Cells,” (student learn how to use a microscope to understand cellular biology and scale, then collect samples in nature to study under magnification), “Simple Machines” (students use tools such as levers, inclined planes, wheel and axels, wedges to safely accomplish tasks in the park, such as transporting or lifting heavy objects like logs and stones; they understand how outdoor play such as sledding and rock climbing depend on these concepts, including work, force and distance) and “Canoeing” (student collect and study samples of aquatic life, such as macro-invertebrates, and learn how safely use a canoe). Students enrolled in the intervention participated in a variety of these programs, dependent on factors including environmental educator availability and weather.

### 2.3. Study Area

The target population and geographic focus of this project is children attending schools within a two-mile radius of The Urban Ecology Center’s new Menomonee Valley location at 3700 W. Pierce Street in Milwaukee, WI. This area includes some of the densest neighborhoods in Wisconsin. According to US Census 2000, an estimated 130,000 people live in the target area of the Menomonee Valley UEC, with 45% living below the poverty line; 73 schools serve more than 30,000 students, 80% of whom qualify for the federal free or reduced lunch program. Students are predominantly (62%) of Hispanic heritage.

### 2.4. Study Design, Recruitment and Sample

This project uses a quasi-experimental pre-post design to evaluate the impact of programming on measures of interest. Six elementary schools within the study area were selected for participation based on their interest in the NEEP program. Initially the project team considered a design that would enroll and randomize 5th grade classrooms at neighboring schools into intervention and control groups. However, early feedback from schools indicated that providing both control and intervention classrooms within a single grade level would dissuade them from participating in the study. Schools indicated that they needed to offer consistent opportunities across a grade level and were not willing to deny one 5th grade class a program that another 5th grade class would receive. As a result, the project team decided to open up the participation to both 5th and 6th grade classrooms so that schools could allow one grade level to be the intervention group and one to be the control group. Schools determined which grade level would receive programming and which would not. Schools were advised against enrolling control group students in other outdoor, nature-based programs during the duration of the study.

**Figure 2:**
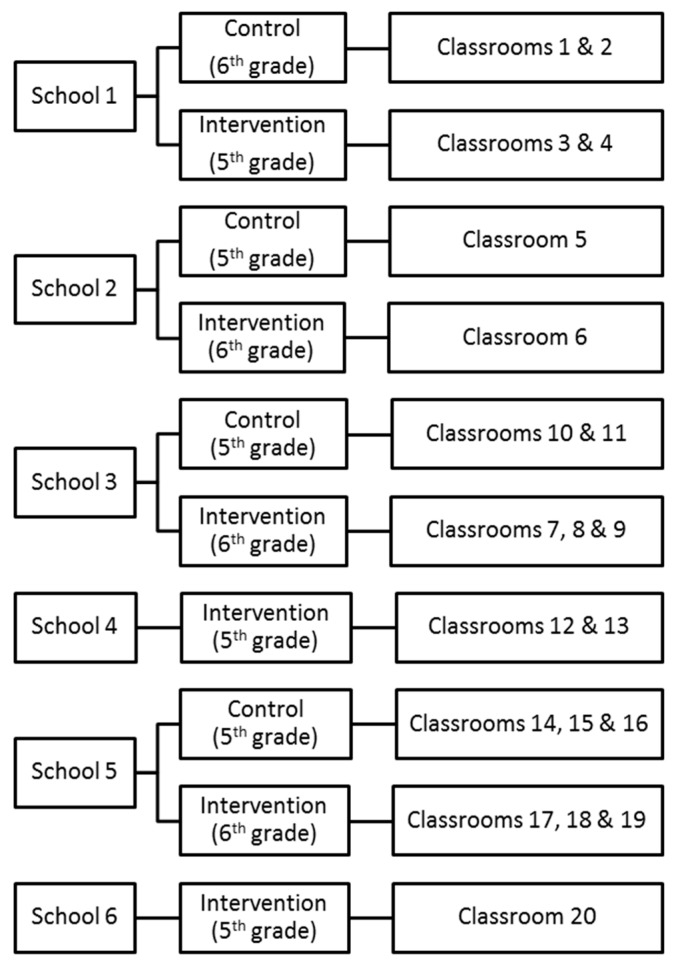
Study Design.

The Menomonee Valley branch of the Urban Ecology Center began looking for schools to partner as part of their NEEP program during the summer of 2012 for the 2012–2013 school year. The Branch Manager of the Menomonee Valley Urban Ecology Center and the Environmental Education Program Manager met with principals and teachers at the schools to discuss the NEEP program and also share the opportunity to have 5th and 6th grade classrooms participate in the More Than A Pretty Place project. Schools agreeing to participate were offered a discounted rate for NEEP programming for one year. Contracts and incentive reductions were negotiated school by school with the Branch Manager, Environmental Education Program Manager, and the Senior Director of Education and Strategic Planning based on the number of classrooms to be enrolled. Individual students did not receive an incentive for participating in the study. Of 12 eligible schools in the area, four declined to participate in the NEEP program. Of the remaining eight schools, six elected to participate in NEEP and the study, one chose to participate in NEEP but declined to participate in the study, and one decided to participate in NEEP but to send 3rd grade classrooms, thus making them ineligible for the study. The demographic composition of schools in the study (74%–99% Hispanic/Latino) is very similar to the demographics of other eligible schools in the area (75%–97% Hispanic/Latino), with the exception of the school that elected not to participate in the study (40% Hispanic/Latino; 42% White/Caucasian). In two cases, schools were enrolled in the project without providing a control group for the study due to the determination that all student responses were of value. Figure 2 displays the study design and classroom enrollment for the six participating schools. Baseline data were collected by survey from October 2012–January 2013 for all classrooms before the intervention groups at each respective school engaged in NEEP programming. Follow-up data collection was undertaken in April and May of 2013 after each school had completed participation in the program. A total of 362 students completed pre-surveys. Due to absences, changes of classrooms, moves outside of the school, and missing information needed to link pre and post surveys, the final sample of matches pre-post surveys is 255.

### 2.5. Measures

The survey instrument administered pre and post participation in programming was developed through an iterative process among the project team, based on existing measures and including new measures. Interviews with environmental educators and two small pilot tests were used to refine the survey instrument and assess difficulties with language, the iPad and the survey application technology used to administer the survey. Of interest to the current paper, student demographics were assessed (age, sex, race, ethnicity). Children’s attitudes toward outdoor play in nature were assessed using the Attitudes toward Outdoor Play scales [39]. One scale measures perceived benefits of outdoor play in nature, while the other measures fears of outdoor play in nature. In addition, students were asked to respond to the following statement with a yes or no: “I know a place in my neighborhood where I can go to play outside in nature.” This statement is intended to measure knowledge of places nearby that are available as locations for outdoor play. Finally, we asked students how often they visit a natural area each month, and how many times they have visited the Urban Ecology Center in the past year, NOT as part of the program offered by their schools (never/one time/two times/more than two times). Additional measures were assessed by the survey but are not discussed in this paper. A combination of student birth date, sex, and race/ethnicity were used to match baseline and follow up surveys, as names were not collected to protect student anonymity.

### 2.6. Data Collection

Teachers in fifth and sixth grade classrooms were contacted to arrange a convenient time for data collection. Parental consent forms were provided in English and Spanish to teachers for distribution to and retrieval from students’ parents. Student assent was obtained in the classroom at the time of the data collection. The Urban Ecology Center evaluation coordinator (EC) led data collection and all team members provided additional support as assistants in the classroom, as necessary.

The number of participants in classrooms ranged from five to forty. The EC explained the purpose of the survey to the students, and informed them that they would be taking the surveys anonymously. The EC then walked through the assent form with the students, and showed them where to print and sign their names. Surveys were administered on iPads with the use of QuestionPro software and the SurveyPocket iPad application. When all students had completed their assent forms, each student was given an iPad. The EC directed students on how to turn on the iPads and find the Survey Pocket application used to take the survey. Because of discoveries during the pilot phase, non-essential iPad features were disabled to ensure that students did not become distracted. In addition, although images were included in the electronic administration of the survey, there were occasions when images would not load on the iPads. When this occurred, the EC would distribute paper copies of the images for the students to consider as they answered the questions. The survey could be taken in either English or Spanish. The EC walked through the first few questions on age, sex, and grade level with the students to demonstrate how to use the survey application and reminded students to keep their hands and eyes on their own surveys to maintain confidentiality. Students were then instructed to begin the survey. The EC and assistant were available to assist students with technical difficulties. Permission was obtained from Milwaukee Public Schools to conduct research, and the study was approved by the Institutional Review Board at The Medical College of Wisconsin. 

### 2.7. Analysis Approach

Student baseline and follow up responses were matched using a combination of the student’s birth date, sex, and race/ethnicity. In some cases, students took only the baseline or follow up survey, or did not indicate a birth date, sex, or race ethnicity. In these cases, the responses were dropped from the dataset. Descriptive statistics were used to summarize information about the study population, and bivariate analyses compared the control and intervention groups. Paired t-tests were used to compare scores on ATOP-benefits and ATOP-fears scales, as well as individual items. McNemar’s test was used to examine changes in knowledge of a place to play outside in nature. The exact Wilcoxon signed-rank test was used to assess change in reported visits to nature and to the Urban Ecology Center. Multilevel linear, logistic or proportional odds ordinal logistic regression modelling, as appropriate for each outcome, was used to predict a student’s time 2 score based on his/her time 1 score, an indicator variable for the intervention group, and two demographic characteristics (sex, ethnicity).

For the continuous outcomes, the models followed the following form:

(1) $$y_{ijk}=\beta_{0}+\beta_{1}{\left(nonhispanic\right)}_{ijk}+\beta_{2}{\left(female\right)}_{ijk}+\beta_{3}{\left(time1score\right)}_{ijk}+\beta_{4}{\left(intervention\right)}_{ijk}+\theta_{k}+u_{jk}+e_{ijk}$$

where:

$$\theta_{k}\sim N\left(0,\sigma_{\theta }^{2}\right)$$

$$u_{jk}\sim N\left(0,\sigma_{u}^{2}\right)$$

$$e_{ijk}\sim N\left(0,\sigma_{e}^{2}\right)$$

This equation is a three level, random intercept model where the outcome ($y_{ijk})$ is the time 2 score for each student (i) nested within a classroom (j) and a school (k), and is explained by the fixed intercept ($\beta_{0})$ which is the average time 2 score for the reference group, the parameters (β) for the variables of interest (non-Hispanic, female, pre-score, and intervention), two random intercept terms that reflect the variation at classroom ($u_{jk}$) and school ($\theta_{k}$) levels, and the residual variance ($e_{ijk})$. Variation is summarized in three variance terms that estimate the between-individual ($\sigma_{e}^{2})$, between-classroom ($\sigma_{u}^{2}$), and between-school ($\sigma_{\theta }^{2}$) variation. The intraclass correlation coefficient (ICC), a measure of agreement of measures within a group, was calculated for both schools and classrooms. For binary and ordinal outcomes the models were generalized by including a logit link-function to obtain multilevel logistic and proportional odds models. Analyses were performed during 2013 and 2014 using STATA SE/12, including the xtmixed, xtmelogit, and meologit commands [40].

## 3. Results and Discussion

### 3.1. Descriptive and Bivariate Statistics

The final pre-post dataset includes 157 intervention and 98 control group participants (n = 255). Table 1 provides a description of the demographics of the study participants, as well as a tabulation of the number of program visits experienced by the intervention group. 

**Table 1:** Sample characteristics.

Variable	Intervention n (%)	Control n (%)	Total n (%)
*Sex*			
Male	70 (44.6%)	50 (51.0%)	134 (52.6%)
Female	87 (55.4%)	47 (48.0%)	120 (47.1%)
Missing		1 (1.0%)	1 (0.4%)
*Race/ethnicity*			
Hispanic/Latino	144 (92.3%)	82 (83.7%)	226 (89.0%)
Non-Hispanic/Latino	12 (7.7%)	16 (16.3%)	28 (11.0%)
*Age*			
10	12 (7.6%)	12 (12.4%)	24 (9.4%)
11	62 (39.5%)	53 (54.6%)	116 (45.5%)
12	70 (44.6%)	30 (30.9%)	100 (39.2%)
13	13 (8.3%)	2 (2.1%)	15 (5.9%)
*Grade*			
5th	55 (35.0%)	75 (76.5%)	130 (51.0%)
6th	102 (65.0%)	23 (23.5%)	125 (49.0%)
*School*			
School 1	27 (17.2%)	23 (23.5%)	50 (19.6%)
School 2	17 (10.8%)	12 (12.3%)	29 (11.4%)
School 3	39 (24.8%)	26 (26.5%)	65 (25.5%)
School 4	23 (14.7%)	0 (0%)	23 (9.0%)
School 5	46 (29.3%)	37 (37.8%)	83 (32.6%)
School 6	5 (3.2%)	0 (0%)	5 (2.0%)
*Number of visits*			
One	51 (32.5%)		51 (32.5%)
Two	89 (56.7%)	n/a	89 (56.7%)
Three	17 (10.8%)		17 (10.8%)

Table 2 presents aggregate pre-scores and post-scores for the intervention and control groups for each of the measures examined, in addition to the change in the measure and whether or not the pre and post scores differed significantly. 

**Table 2:** Pre, post and change in scores for intervention and control group.

	Intervention Group (n = 157)				Control Group (n = 98)			
**Measures**	**Before (mean)**	**After (mean)**	**Change**	***p*−Value**	**Before (mean)**	**After (mean)**	**Change**	***p*−Value**
**ATOP-benefits**	3.19	3.17	−0.02	0.61	3.23	3.21	−0.02	0.71
Playing outside in nature helps me think more clearly.	3.08	3.15	0.07	0.32	3.08	3.10	0.02	0.82
Playing outside in nature makes me healthier.	3.41	3.43	0.01	0.85	3.43	3.53	0.10	0.15
When I’m angry, playing outside in nature calms me down.	3.06	2.95	−0.11	0.20	2.89	2.88	−0.01	0.93
I learn new things when I play outside in nature.	2.95	2.94	−0.01	0.92	3.12	3.07	−0.05	0.56
I feel like I have freedom when I play outside in nature.	3.37	3.33	−0.04	0.58	3.49	3.38	−0.11	0.15
I like to make up games when I’m outside in nature.	3.16	3.08	−0.08	0.34	3.16	3.04	−0.12	0.15
I like to explore new places outside in nature.	3.21	3.32	0.10	0.16	3.30	3.40	0.09	0.20
**ATOP-fears**	2.15	1.92	−0.24	0.00 **	2.21	2.10	−0.11	0.10
I am afraid of getting lost outside in nature.	2.30	1.95	−0.35	0.00 **	2.23	2.09	−0.13	0.25
I don’t like playing outside in nature because there are strangers.	2.10	1.92	−0.18	0.05 *	2.19	2.03	−0.16	0.09
I am afraid of wild animals or insects outside in nature.	2.14	1.90	−0.24	0.00 **	2.16	2.09	−0.07	0.47
I am afraid of getting hurt if I play outside in nature.	1.92	1.75	−0.16	0.04 *	2.04	2.00	−0.04	0.66
I don’t like playing outside in nature because there are people with drugs.	2.30	2.07	−0.24	0.02 *	2.45	2.31	−0.14	0.29
**Measures**	**Before (perc)**	**After (perc)**	**Change**	***p*−Value**	**Before (perc)**	**After (perc)**	**Change**	***p*−Value**
**Visit urban ecology center (per month)**								
Never	84.7%	62.2%	−22.53		76.0%	77.6%	1.51	
Onetime	7.6%	7.1%	−0.59		12.5%	16.3%	3.83	
Two times	1.9%	13.5%	11.55		5.2%	4.1%	−1.13	
More than two times	5.7%	17.3%	11.58	0.00 **	6.3%	2.0%	−4.21	0.50
**Visit nature (per month)**								
Never	9.6%	10.3%	0.71		7.2%	1.0%	−6.18	
One time	21.0%	15.4%	−5.64		15.5%	18.8%	3.29	
Two times	16.6%	10.3%	−6.30		12.4%	12.5%	0.13	
More than two times	52.9%	64.1%	11.23	0.12	65.0%	67.7%	2.76	0.18
**Know a place to play**								
Yes	71.3%	84.1%	12.76		80.6%	86.7%	6.09	
No	28.0%	15.9%	−12.11	0.00 **	19.4%	13.3%	−6.12	0.00 **

Notes: ** *p* < 0.01, * *p* < 0.05.

The ATOP-fears score, and scores for each item composing the scale, were significantly reduced in the intervention group, but not in the control group, providing evidence in support of the intervention’s effectiveness at reducing fears of outdoor play in nature among study participants. In addition, the number of participants who agreed with the statement “I know a place in my neighborhood where I can go to play outside in nature” increased significantly in both groups, with a larger increase in the intervention group. In addition, the intervention group reported significantly more frequent visits to the Urban Ecology Center following the intervention. The average ATOP-benefits scores and frequency of visiting a natural area did not change significantly for either the intervention or control group.

### 3.2. Multilevel Regression Models

Table 3 shows the results of the multivariate analyses, which are mostly consistent with the bivariate results.

**Table 3:** Multilevel multivariable regression models measuring the impact of the neighborhood environmental education project.

Variables	ATOP-Benefits	ATOP-Fears		Know a Place	Visit Nature	Visit Urban Ecology Center
**Fixed effects**	*Reported as coefficients with 95% confidence intervals*			*Reported as odds ratios with 95% confidence intervals*		
Baseline Score $\left(\beta_{1}\right)$	0.541	0.410		4.68	Never Referent	Never Referent
	[0.423, 0.659] **	[0.310, 0.510] **		[2.20, 9.95] **		
					Once 2.27	Once 1.86
					[0.85, 6.01]	[0.76, 4.55]
				
					Twice 2.98	Twice 1.26
					[1.03, 8.66] *	[0.23, 6.87]
				
					More than 2 times	More than 2 times
					9.22	3.10
					[3.62, 23.46] **	[1.07, 8.96] *
Female $\left(\beta_{2}\right)$	0.039	0.097		1.06	1.27	0.63
	[−0.152, 0.074]	[-0.036, 0.231]		[0.50, 2.26]	[0.72, 2.24]	[0.34, 1.05]
Non-Hispanic $\left(\beta_{3}\right)$	−0.005	−0.231		5.96	1.41	1.73
	[−0.185, 0.175]	[−0.437, −0.024] *		[0.75, 47.15]	[0.52, 3.88]	[0.74, 4.05]
Intervention $\left(\beta_{4}\right)$	−0.045	−0.170		1.16	0.84	3.18
	[−0.198, 0.108 ]	[−0.304, −0.037] *		[0.53, 2.52]	[0.44, 1.58]	[1.41, 7.19] **
**Random effects **		*Reported as variance*					
Classroom (variance, $\sigma_{e}^{2})$	0.013 (0.009)	0.000 (0.000)		0.000 (0.000)	0.068 (0.166)	0.332 (0.277)
School (variance, $\sigma_{e}^{2})$	0.000 (0.000)	0.005 (0.007)		0.000 (0.000)	0.000 (0.000)	0.000 (0.000)
Residual variance $\left(\sigma_{e}^{2}\right)$	0.185 (0.017)	0.263 (0.024)		--	--	--
**Diagnostics**						
ICC (classrooms in schools)	0.067	0.018		0.000	0.020	0.092
ICC (school)	0.000	0.000		0.000	0.000	0.000

Notes: ** *p* < 0.01; * *p* < 0.05.

When controlling for sex, ethnicity, and pre-scores, the intervention was found to significantly reduce fears of outdoor play in nature and increase frequency of visits to the Urban Ecology Center. The odds of visiting the Urban Ecology Center outside of school programs were 3.18 times higher in the intervention group as opposed to the control group. The positive change in knowledge of a place to play outside in nature was no longer significant. ICC estimates, representing the correlation between post-scores among children in the same classrooms and schools, suggest that classroom and school effects compose approximately 7% of the total residual variance for ATOP-benefits, 2% of the total residual variance for ATOP-fears (at each of the two levels), 2% of the variance in visits to nature, and 9% of the variance in visits to the Urban Ecology Center. Classroom and school effects were not important in explaining the variance of knowledge of a place to play outside in nature. 

The objective of this study was to assess the impact of the Urban Ecology Center’s work—specifically, the Neighborhood Environmental Education Project (NEEP)—on attitudes toward outdoor play in nature among children. Fears of outdoor play in nature were reduced in the intervention group, but not the control group. Students in the intervention group reported increased frequency of visits to the Urban Ecology Center. The proportion of students who acknowledged that they knew of a place to play outside in nature increased significantly in both groups in unadjusted analyses, and no effect was found for the intervention when controlling for other factors. Because a brownfield was converted into a 24 acre park in the neighborhood during this time, and we anticipate that students in the control group may have learned about this development through residence in the area or from classmates who visited the Urban Ecology Center, this finding is not surprising. It is interesting to note that a larger change in knowledge was reported in the intervention group, although this difference was not statistically significant. No effect of the intervention was detected on attitudes toward the benefits of outdoor play in nature, or the frequency of nature visits.

Study limitations include the non-random assignment of classrooms to intervention and control groups, uneven participation of 5th and 6th grade classrooms in the intervention, and the lack of control classrooms in two schools. These limitations in the study design were necessary to secure school participation in the study and are a common concern in community engaged intervention research. In addition, students who participated in the intervention received a variety of programming, dependent on logistical factors; future work should interrogate the different impacts of different types of programs. Although schools were asked not to enroll control group students in alternative nature-based environmental education programs, it is difficult to confirm whether this occurred. However, if control group participants engaged in similar programming, it would bias study findings toward a null result. We were unable to assess differences between students whose parents/guardians provided consent to participate in the study and those who did not, as school records necessary to do so are confidential. Finally, the study relies on children’s self-report, which is subject to error, and it is possible that there are other factors that we were unable to asses that could have affected study results. 

## 4. Conclusions

Urban parks have tremendous potential value for addressing health problems in urban neighborhoods, including through their use as active outdoor play spaces. However, the mere existence of a park may not automatically relay benefits to the community. Urban parks can be magnets for socially deviant behavior, including crime and vandalism. Parks must be properly leveraged to act as an amenity that positively shifts the health status of residents in low SES neighborhoods. A recent report funded by the Centers for Disease Control and Prevention (CDC) and the Robert Wood Johnson Foundation confirmed this assertion, noting “Even if a park system offers varied spaces for physical activity, not everyone will know how to take advantage of them… Good programming can increase park use many times over, make activity more enjoyable and increase its benefit to health and fitness” [41]. The Urban Ecology Center activates urban green spaces through programming, events and neighborhood-wide participation in environmental education, recreational activities, and other programming, and this study examined the impact of one of these programs on knowledge and attitudes regarding outdoor play in nature. 

Our findings indicate a potentially important role for environmental education in addressing fears that may dissuade children from engaging in active outdoor play in natural areas. The intervention reduced fears associated with outdoor play in nature, potentially reducing an important barrier to physical activity in natural environments. Our findings have implications for programs that intend to connect children with nature and increase their levels of active outdoor play, particularly in urban and Hispanic/Latino communities in the USA. Additional research is needed to examine the long term effect of environmental education programming on levels of active outdoor play. In particular, future work should explore longitudinal changes in activity patterns that may take place through an extended engagement in outdoor programming such as that offered by the Urban Ecology Center. Engaging children in outdoor environmental education to enhance long term mental and physical health is a promising line of work that deserves additional attention.
